# Distinct 3′ UTRs regulate the life-cycle-specific expression of two *TCTP* paralogs in *Trypanosoma brucei*

**DOI:** 10.1242/jcs.206417

**Published:** 2018-05-10

**Authors:** Borka Jojic, Simona Amodeo, Irina Bregy, Torsten Ochsenreiter

**Affiliations:** 1 Institute of Cell Biology, University of Bern, Bern 3012, Switzerland; 2 Graduate School for Cellular and Biomedical Sciences, University of Bern, Bern 3012, Switzerland

**Keywords:** TCTP, TPT1, *Trypanosoma brucei*, Mitochondria, Acidocalcisomes, Growth defects, Gene expression regulation, 3′UTR

## Abstract

The translationally controlled tumor protein (TCTP; also known as TPT1 in mammals) is highly conserved and ubiquitously expressed in eukaryotes. It is involved in growth and development, cell cycle progression, protection against cellular stresses and apoptosis, indicating the multifunctional role of the protein. Here, for the first time, we characterize the expression and function of TCTP in the human and animal pathogen, *Trypanosoma brucei*. We identified two paralogs (*TCTP1* and *TCTP2*) that are differentially expressed in the life cycle of the parasite. The genes have identical 5′ untranslated regions (UTRs) and almost identical open-reading frames. The 3′UTRs differ substantially in sequence and length, and are sufficient for the exclusive expression of *TCTP1* in procyclic- and *TCTP2* in bloodstream-form parasites. Furthermore, we characterize which parts of the 3′UTR are needed for *TCTP2* mRNA stability. RNAi experiments demonstrate that TCTP1 and TCTP2 expression is essential for normal cell growth in procyclic- and bloodstream-form parasites, respectively. Depletion of TCTP1 in the procyclic form cells leads to aberrant cell and mitochondrial organelle morphology, as well as enlarged, and a reduced number of, acidocalcisomes.

## Abstract (precis)

**Summary:** *T. brucei* has two *TCTP* genes that are differentially expressed during the parasite life cycle owing to their different 3′UTRs. TCTP also has a role in regulating cell growth and morphology.

## INTRODUCTION

Since the time of its discovery ∼30 years ago (Chitpatima et al., 1988), TCTP (also known as TPT1 in mammals) has continuously attracted research interest due to the high conservation among eukaryotes and involvement in a large number of biological processes (Bommer and Thiele, 2004). TCTP is expressed in most eukaryotes and, in contradiction with the assigned name, not exclusively in cancerous tissues but in all the tested animal and plant tissues, with preference towards the mitotically active tissues and less in the post-mitotic tissues, such as the brain (Berkowitz et al., 2008; Hinojosa-Moya et al., 2008; Sanchez et al., 1997; Thiele et al., 2000). In humans and rabbits the gene encoding for TCTP is transcribed in two mRNAs (named *TCTP* mRNA1 and *TCTP* mRNA2), which contain the same 5′ untranslated region (UTR) and open-reading frame (ORF) but different lengths of their 3′UTRs, generated by alternative polyadenylation (Thiele et al., 2000). The two mRNAs are co-expressed but they differ in the ratio of their expression, with the shorter mRNA1 being more abundant. The functional significance of the existence of these two transcripts has not yet been identified. The predicted secondary structure elements of TCTP consist of three α-helices and 11 β-stands and, to date, a microtubule-binding, a Ca^2+^-binding and two TCTP signature domains (TCTP1 and TCTP2) have been mapped (Bommer and Thiele, 2004). Despite the many years and number of research, an exact molecular function of TCTP has not yet been elucidated in any of the studied organisms. However, different studies have shown that TCTP is involved in many biological processes depending on the type of the cells/tissue, most notably growth and development, apoptosis, protection against cellular stresses and the cell cycle (Berkowitz et al., 2008; Cao et al., 2010; Chan et al., 2012b; Chen et al., 2007; Gnanasekar et al., 2009; Gnanasekar and Ramaswamy, 2007; Hsu et al., 2007; Mak et al., 2001). Moreover, several interacting/binding partners, such as elongation factor eEF-1δ (Langdon et al., 2004), tubulin (Tuynder et al., 2002), Ca^2+^ (Haghighat and Ruben, 1992) and Na^+^/K^+^-ATPase (Jung et al., 2004) have been identified. The protein is primarily localized in the cytosol; however, in mammals and yeast it has been shown that TCTP can localize to the nucleus or mitochondria, respectively, when cells are exposed to certain stress conditions (Diraison et al., 2011; Rid et al., 2010; Rinnerthaler et al., 2006).

Here, for the first time, we study the expression and function of TCTP in the unicellular parasite *Trypanosoma brucei*. Trypanosomes are single-celled flagellated protozoa with a complex structured mitochondrial genome known as the kinetoplast (Stuart et al., 2008; Vickerman, 1976). They alternate their life cycle between the bloodstream of a mammalian host (bloodstream form, BSF) and different compartments of the tsetse fly *Glossina* spp., including the midgut where they proliferate as procyclic forms (PCFs). They undergo several differentiation steps in order to ensure survival in the different environments (Vickerman, 1965). The differentiation steps are accompanied by extensive gene regulation that enables the parasite to survive in varying host environments characterized by different energy sources, temperature and pH. Owing to the continuous polycistronic transcription, unlike other eukaryotes, the regulation of individual gene expression occurs mainly at the post-transcriptional level through *cis*- and *trans*-acting elements (Clayton, 2013; Haile and Papadopoulou, 2007). The *cis*-acting elements are mainly motifs in the 3′UTRs, which confer repression, instability or increased translation of the mRNAs during the transition of the parasites from one form to another (Hehl et al., 1994; Hotz et al., 1997; MacGregor and Matthews, 2012).

In trypanosomes, the cell cycle is strongly connected to the parasite asymmetrical elongated morphology, maintained by a sub-pellicular microtubule corset (Hemphill et al., 1991). The microtubules in the corset are interconnected with each other and the plasma membrane, and display an intracellular polarity with their plus-ends at the posterior of the cell (Hemphill et al., 1991; Robinson et al., 1995). During cell division, the microtubular corset does not break down. Instead the newly synthesized microtubules are integrated into the old microtubule array making its inheritance between the daughter cells semiconservative (Sherwin and Gull, 1989). A number of single-copy organelles with a precisely defined cellular position are present in trypanosomes (i.e. the flagellum, the nucleus, the kinetoplast, the mitochondrion, the basal body and the Golgi). During the cell cycle their duplication follows a specific temporal and spatial order to allow for proper cell division (McKean, 2003). Similar to what is found in other eukaryotes, the cell cycle pattern in trypanosomes follows a G1, S, G2, mitosis (M) and cytokinesis phase (Hammarton, 2007; Woodward and Gull, 1990). However, different from animals, where cytokinesis requires the formation of an actomyosin contractile ring, in trypanosomes the presence of actin is not required for cytokinesis and it instead depends on the proper separation and formation of the microtubule corset (García-Salcedo et al., 2004). Moreover, the placement of the cleavage site is asymmetrical (Wheeler et al., 2013). There are two important consequences of this asymmetry: (1) different zones of the dividing cell body undergo different growth and morphogenetic events, and (2) the resulting two daughter cells inherit different portions of the existing cell body. One daughter cell receives the posterior part of the mother cell with the new flagellum, while the other daughter inherits the anterior part with the old flagellum. The middle zone produces a novel anterior end for the new-flagellum daughter cell and a new posterior end for the old-flagellum daughter cell (Wheeler et al., 2013).

Twenty five years ago a Ca^2+^-binding protein was isolated from *T. brucei* (Haghighat and Ruben, 1992) that showed significant similarity to the TCTP from mammalian cells, which was later also confirmed by a phylogenetic study (Hinojosa-Moya et al., 2008). Furthermore, a number of high-throughput studies have shown expression and localization of a *T. brucei* TCTP (Aslett et al., 2010). Here, we present, for the first time, data on the identification of two *TCTP* paralogs in *T. brucei*, and carefully characterize the differential expression, mechanism of differential regulation, localization and TCTP-loss phenotypes in blood- and insect-form *T. brucei.*

## RESULTS

### Bioinformatics analysis of *TCTP*

**Fig. 1.**
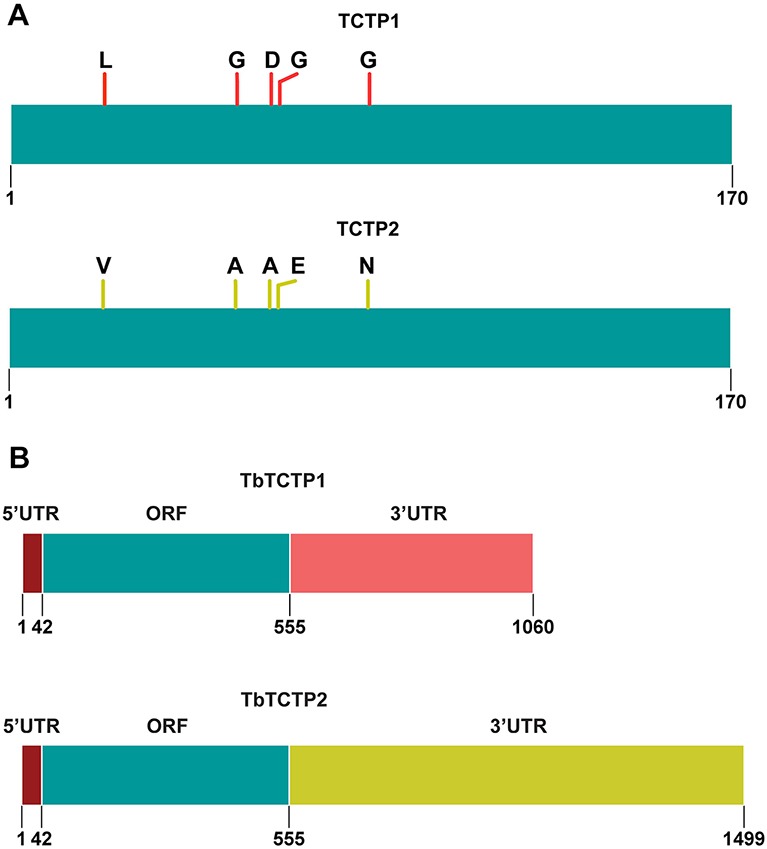
**TCTP1 and TCTP2 in *T. brucei*.** (A) Depiction of the TCTP1 and TCTP2 amino acid sequence. The proteins differ in only five amino acids at the positions 23, 54, 62, 64 and 85. (B) Representation of the two *TCTP* paralog genes in *T. brucei* (*TCTP1* and *TCTP2*). Depicted are the 5′UTRs (dark red), the open reading frame (ORF, blue) and the 3′UTRs (pink, dark yellow). The 5′UTRs are identical in sequence.

We have identified a paralog of the previously described *TCTP* homolog in *T. brucei* (Hinojosa-Moya et al., 2008). The two genes are tandemly arrayed on chromosome eight and we named them *TCTP1* (Tb927.8.6750) and *TCTP2* (Tb927.8.6760). Phylogenetic analysis of the TCTP protein sequence confirms the very conserved primary structure throughout the eukaryotic supergroups (Hinojosa-Moya et al., 2008). Most of the currently sequenced Kinetoplastea genomes contain two paralog *TCTP* genes similar to what has been described in *Arabidopsis* and *Dictyostelium* (Hinojosa-Moya et al., 2008). Within the Kinetoplastea, the *TCTP* orthologs show up to 80% sequence similarity, while it exceeds 95% in the paralogs of this group (Fig. S1). In several Kinetoplastea, including *Leishmania major* and *Crithidia fasciculata*, the paralogs are identical in the amino acid sequence. In *T. brucei*, the paralogs contain five amino acid changes of which three are non-conservative (L23V, G54A, D62A, G64E, G85N; Fig. 1A). The *TCTP* sequence conservation between Kinetoplastea and other eukaryotes is up to 35% and includes the proposed microtubule- binding, Ca^2+^-binding and TCTP domains (Fig. S2). Both genes have an identical 5′UTR and ten nucleotide changes in the ORF, leading to the five changes at the amino acid level. However, the 3′UTRs of *TCTP1* and *TCTP2* differ drastically in sequence and length (Fig. 1B).

### *TCTP* paralogs expression in different life cycle stages of *T. brucei*

**Fig. 2.**
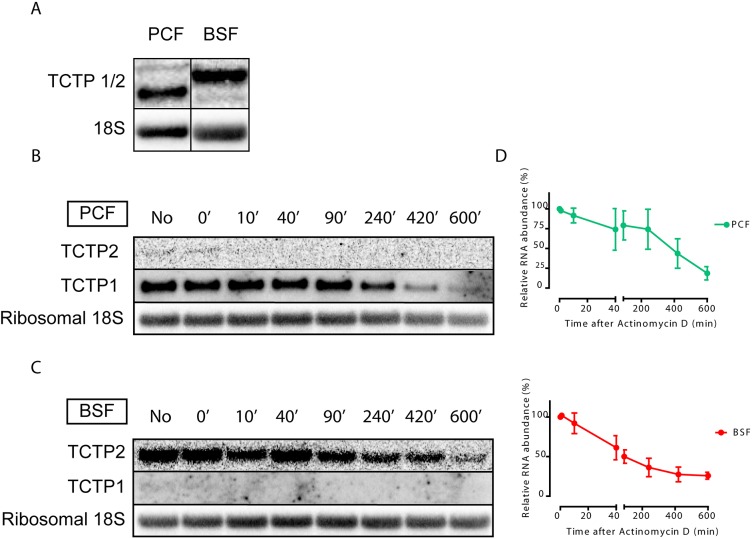
***TCTP* mRNA expression is developmentally regulated in *T. brucei*.** (A) *TCTP* mRNA expression in BSF and PCF trypanosomes. (B,C) Northern blot analysis of *TCTP1* and *TCTP2* mRNA from BSF and PCF trypanosomes. The RNA was collected from cultures incubated with ActD (10 µg/ml) for indicated time (minutes). Ribosomal 18S RNA is used as a loading control. (D) Quantification of the relative RNA abundance of *TCTP1* in PCF and *TCTP2* in BSF trypanosomes. Three independent experiments were performed for each time point. Error bars represent standard deviation. Values are normalized to 18S rRNA. mRNA abundance in untreated cells was assigned as 100%. Half-lives were calculated to be 217 min in the PCF and 51 min in the BSF.

**Fig. 3.**
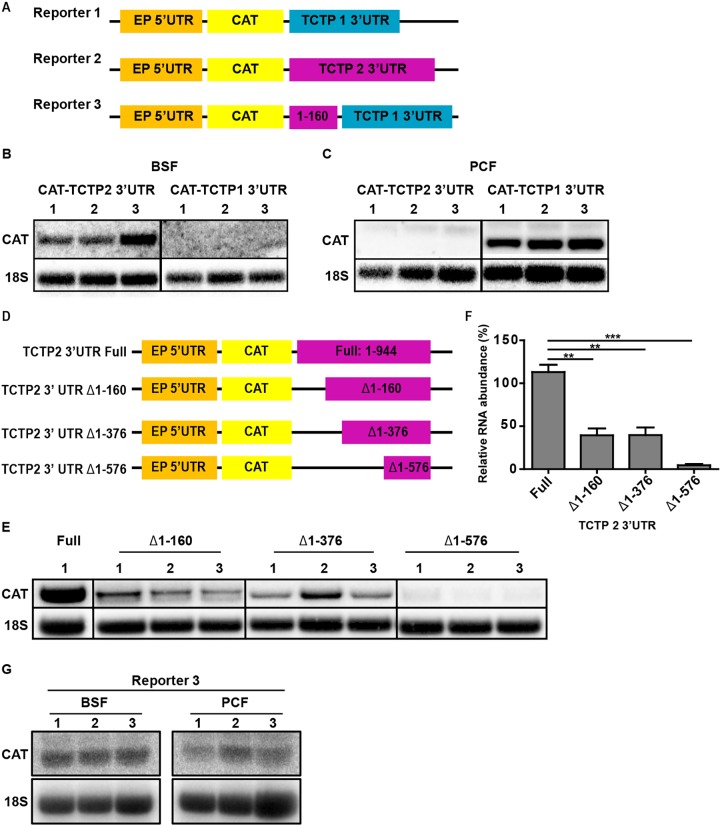
**Role of the distinct *TCTP* 3′UTRs in life-cycle-specific mRNA expression.** (A) Schematic representation of the three reporter constructs linking the early procyclin (EP) 5′UTR (orange) and *CAT* ORF (yellow) to the *TCTP1* (blue, reporter 1) or *TCTP2* (magenta, reporter 2) 3′UTR or the chimera of the 5′ 1–160 TCTP2 UTR followed by the full-length TCTP1 3′UTR (magenta and blue, reporter 3). Each construct was transfected in BSF and PCF trypanosomes. (B,C) Total RNA from three clones per condition was isolated and *CAT* mRNA expression was analyzed by northern blotting, probing for the *CAT* ORF. Ribosomal 18S was used as a loading control. (D) Schematic representation of constructs used for these experiments. Full-length or truncated versions of the *TCTP2* 3′UTR (Δ1–160, Δ1–376 and Δ1–576) were linked downstream of the EP procycline 5′UTR and *CAT* ORF. (E,G) Total RNA from three clones per condition was isolated and *CAT* mRNA expression was analyzed by northern blotting, probing for *CAT* ORF. Ribosomal 18S was used as a loading control. (F) Quantification of the relative abundance of *CAT* mRNA. Values were normalized to the level of *CAT* expression with the full-length *TCTP2* 3′UTR. Bars represent s.e.m. (*n*=3). **P<0.01; ***P≤0.001 (one-tailed *t*-test).

When we probed for *TCTP* mRNA expression in BSF and PCF parasites, we found that two different isoforms of the gene were expressed (Fig. 2A). We wondered whether these isoforms represent the different paralogs and probed for the specific *TCTP1* and *TCTP2* 3′UTRs in the BSF and PCF parasites. Northern blot analysis confirmed that *TCTP1* mRNA is expressed in PCF trypanosomes, while the *TCTP2* mRNA is barely detectable in this life cycle stage (Fig. 2B). The opposite is observed for BSF trypanosomes, where *TCTP2* is the predominantly expressed paralog (Fig. 2C). To compare the mRNA stability of the paralogs, we determined the half-life of each transcript in the life cycle stage where it is expressed. For this, we incubated the cells with Actinomycin D (ActD, 10 µg/ml) to stop transcription. Total RNA was collected at the indicated time points after ActD treatment (Fig. 2B,C) and analyzed by northern blotting. 18S ribosomal RNA was used as a loading control. The mean relative mRNA abundance of *TCTP1* and *TCTP2* from eight time points following ActD treatment was measured and showed a continuous decrease. The half-life of *TCTP1* mRNA in the PCF was calculated to be 217 min, while the half-life of *TCTP2* mRNA in the BSF was 51 min (Fig. 2D). To better understand the mechanism of stage-specific expression and the role of the 3′UTRs, we created two constructs in which the *TCTP1* or *TCTP2* 3′UTR is linked to the ORF of a reporter gene (chloramphenicol acetyltransferase, *CAT*; Fig. 3A). Both constructs were transfected in PCF and BSF trypanosomes to integrate into the tubulin locus. Total RNA from three different clones of each life cycle stage was isolated and further analyzed by probing against the *CAT* ORF (Fig. 3B,C). We observed that in PCF trypanosomes, the *CAT-TCTP1* construct was well expressed in all clones, while the *CAT-TCTP2* construct was barely detectable (Fig. 3C). In the BSF cells, the *CAT-TCTP2* was detected in all clones whereas the *CAT-TCTP1* construct was not detectable (Fig. 3B). Thus, the 3′UTRs of *TCTP1/2* are responsible for the differential expression of *TCTP1* and *TCTP2* in PCF and BSF trypanosomes, respectively. We further investigated which part of the *TCTP2* 3′UTR is required for the mRNA stability in BSF trypanosomes. For this, we created three additional *CAT* reporter constructs with different 3′UTR deletions: (1) deletion of nucleotides (nt) 1–160, (2) deletion of nt 1–376, and (3) deletion of nt 1–576 (Fig. 3D). All constructs were separately transfected in BSF trypanosomes, and total RNA was isolated from three clones of each construct. Northern blot analysis was performed by probing for the *CAT* ORF (Fig. 3E) and revealed that deletion of the first 160 nt of the 3′UTR leads to a >50% decrease in mRNA stability, while the additional deletion of ∼200 nt does not change the stability (Fig. 3E,F). Deletion of the entire 3′UTR except the region required for 3′-end processing leads to almost complete loss of the *CAT* signal (Fig. 3E,F). We also tested whether the 5′ 160 nt of the *TCTP2* 3′UTR that are required for proper expression in BSF cells are also sufficient to confer stability in the BSF cells even in the context of the *TCTP1* 3′UTR (Reporter 3, Fig. 3A). Northern blot analysis of three different clones revealed that indeed the 160 nt of the *TCTP2* UTR are sufficient to override the *CAT* reporter destabilization caused by the *TCTP1* 3′UTR (Fig. 3G). Furthermore, when we transfected the chimeric reporter construct in PCF cells, a significant destabilization of the *CAT* mRNA was observed (Fig. 3G).

### Localization of TCTP1 in PCF trypanosomes

**Fig. 4.**
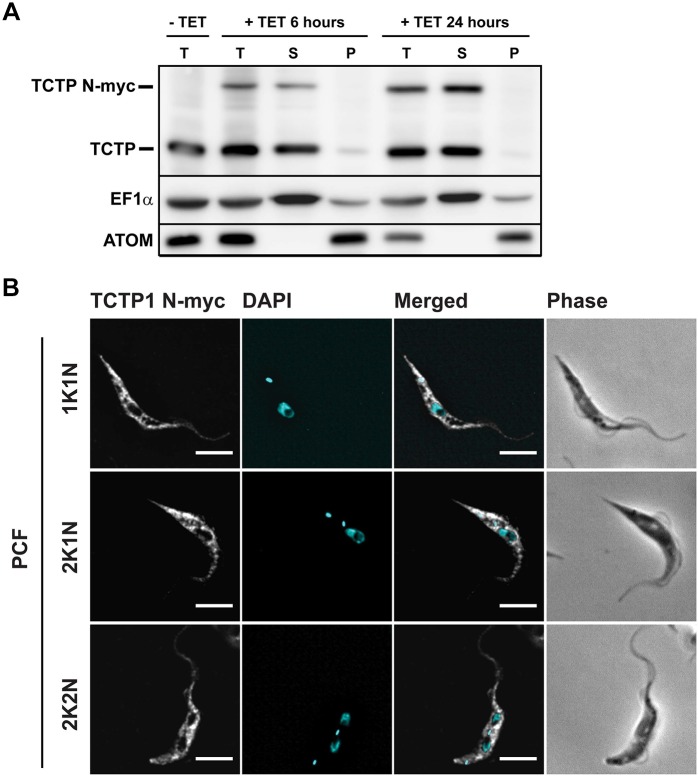
**Localization of the N-terminally tagged TCTP1 in procyclic trypanosomes.** (A) Western blot of digitonin fractions from cells expressing Myc-tagged TCTP1. Total cellular extract (T), the supernatant fraction enriched in cytosolic proteins (S), and the pellet fraction enriched in organelles and organelle-bound proteins (P) were analyzed by western blotting with antibodies against TCTP, ATOM and EF1α. Expression of N-terminally tagged Myc–TCTP1 was induced by incubation with tetracycline (TET) for 6 and 24 h. (B) N-terminally tagged TCTP1 was detected by immunofluorescence microscopy with anti-Myc antibody (white). The DNA was detected with DAPI (cyan). Scale bars: 5 µm. 1K, 2K, number of kinetoplasts; 1N, 2N, number of nuclei.

We focused on TCTP1 in the PCF cells for the further analysis of the localization and function, since many more studies concerning cell/organelle morphology have been undertaken in the insect form of the parasite and PCF parasites grow to much higher densities, simplifying biochemical analyses. In order to localize TCTP1, we produced a polyclonal antibody in rats. In biochemical fractionations of total cell extracts with digitonin, TCTP1 localized to the cytoplasmic fraction (Fig. 4A). Since the antibody did not work in immunofluorescence microscopy, we tagged TCTP1 N- (Myc) and C-terminally (triple HA), and checked for its localization with anti-Myc and anti-HA antibodies. The N- or C-terminally tagged ectopically expressed TCTP1 localized to the cytoplasmic fraction (Fig. 4A; Fig. S3A) and was distributed in the cell in a pattern that is also consistent with a cytoplasmic localization (Fig. 4B; Fig. S3B). Additionally, we found a discernible depletion of TCTP in the nucleus (Fig. 4B; Fig. S3B), and did not detect any change of localization or abundance during the cell cycle (Fig. 4B).

### Depletion of TCTP1 leads to a slow growth phenotype, and affects several organelles and cytokinesis

**Fig. 5.**
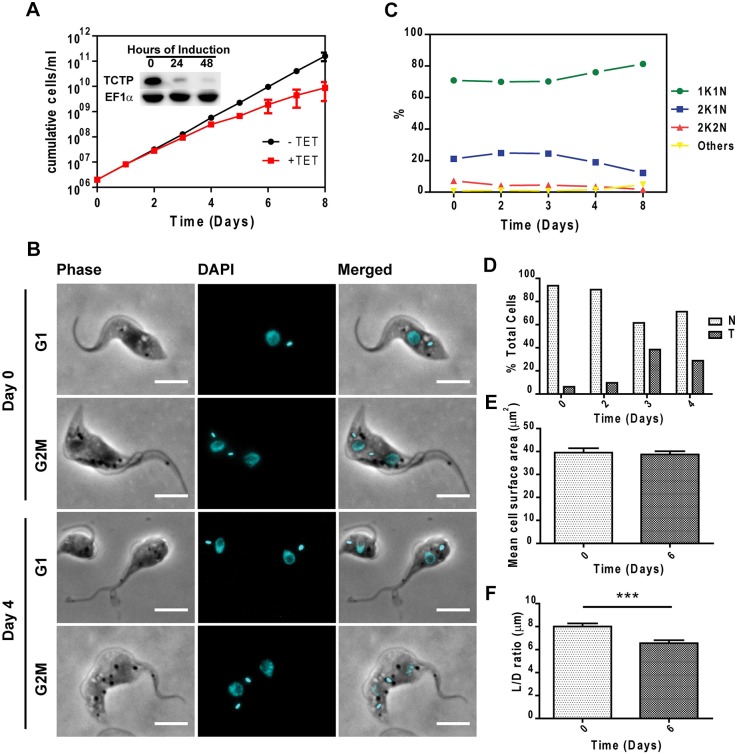
**The effect of TCTP downregulation on the growth and morphology of procyclic trypanosomes.** (A) Growth curve of cells that were induced/uninduced (+Tet/-Tet) for RNAi-based TCTP depletion monitored for 8 days. Inset: western blot showing TCTP depletion upon 24 and 48 h of RNAi induction. EF1α is used as loading control. (B) Representative single-cell images of non-induced and tadpole-like trypanosomes in G1 and G2M stages of the cell cycle from a population of day 4 induced *TCTP1/2* RNAi trypanosomes. (C) Percentage of cells in different cell cycle stages and (D) percentage of cells with tadpole phenotype in a population of TCTP RNAi procyclic trypanosomes (*n*≥250 for each time point). (E) Cell surface area change upon *TCTP1/2* RNAi. (F) Ratio of cell body length L to cell body diameter D in non-induced and induced cells (***P=0.0004, two-tailed student's *t*-test, *n*≥20). Results in E and F are mean±s.d. Scale bars: 5 µm.

To determine the function of TCTP1 in PCF parasites, we depleted *TCTP1/2* mRNA by performing RNAi experiments against the *TCTP1/2* ORF (Fig. 5A). We could show that TCTP protein levels were strongly depleted after 48 h of RNAi induction (Fig. 5A, inset). PCF cells started growing slower at day 3 post induction and continued to do so at least until day 8 (Fig. 5A). We analyzed the karyotype and the morphology of the TCTP-depleted cells by staining for the nucleus and kinetoplast DNA with DAPI, and also acquiring phase-contrast images (Fig. 5B). Upon 8 days of *TCTP1/2* RNAi induction, we observed an increase in the proportion of 1K1N cells from 71% to 81% and a decrease of 2K1N cells from 21% to 12% (Fig. 5C). We noticed an accumulation of cells displaying a ‘tadpole’ morphology (up to 40%, Fig. 5D). These cells are characterized by a distinct shape of the cell body, where the posterior end of the cell is round and enlarged whereas the anterior becomes more slender than in the wild-type situation (Fig. 5B, G1). This change in morphology was confirmed by calculating and comparing the ratio of the cell body length (L) to the cell body diameter through the nucleus (D), as well the cell surface area in the same population of non-induced (*n*=20) and induced cells (*n*=24). While there was no significant change in the mean cell surface area of the induced population (Fig. 5E), a significant reduction in L:D ratio was seen for the cells where TCTP was depleted (Fig. 5F). When we measured tadpoles only (*n*=7), the mean surface area is reduced from an average of 40 µm^2^ to an average of 29 µm^2^. The L:D ratio was also reduced from an average of 8 in the non-induced population to an average of 5.4 in the induced population. This phenotype was observed exclusively in 1K1N cells, indicating that the change in shape is likely a consequence of improper cytokinesis.

**Fig. 6.**
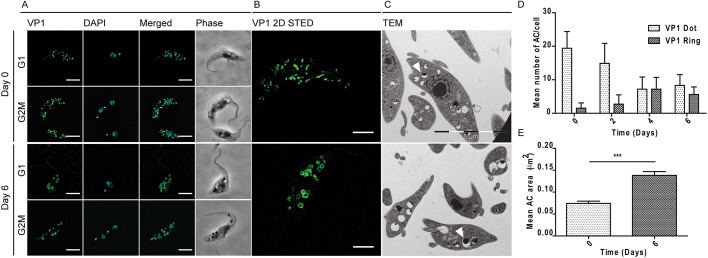
**Morphology of acidocalcisomes following depletion of TCTP.** PCF trypanosomes that were non-induced and induced (day 2, 4 and 6) for *TCTP1/2* depletion were stained for (A) the acidocalcisome marker VP1 (green) and with DAPI (cyan) for the nucleus and kDNA. Cell morphology is shown in the main phase-contrast images (gray). (B) Representative images of acidocalcisomes in single cells acquired by super resolution microscopy. (C) Representative images of acidocalcisome ultrastructure as obtained by transmission electron microscopy. Arrowheads point to examples of acidocalcisomes to demonstrate their size in non-induced and induced cells. (D) Histograms showing the mean±s.e.m. number of dot-like versus ring-like acidocalcisomes per cell after 0, 2, 4 or 6 days of induction (*n*=10). (E) Quantification of mean±s.e.m. acidocalcisome area in TEM sections of cells after 0 and 6 days of induction (*n*=80, one-tailed *t*-test, ***P<0.0001). Scale bars: 5 µm (A,C), 2 µm (B).

**Fig. 7.**
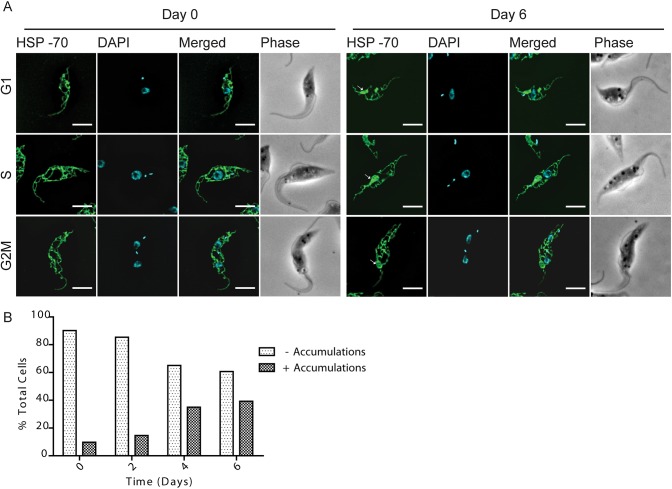
**Effect of TCTP downregulation on mitochondria.** (A) Immunofluorescence images of non-induced (Day 0) and induced (Day 6) procyclic cells stained for mitochondria (HSP-70, green) and with DAPI (nucleus and kDNA, cyan). Phase-contrast images (gray) show cell morphology. Arrows point to mitochondrial accumulations. Representative images of cells in G1, S and G2M cell cycle stages are selected for both non-induced and induced cell populations. (B) Quantification of the percentage of cells with (+) or without (–) mitochondrial accumulations after 0, 2, 4 or 6 days of induction (*n*=160). Scale bars: 5 µm.

Since TCTP has been shown to be a Ca^2+^-binding protein in trypanosomes (Haghighat and Ruben, 1992), we wondered whether depletion of the protein would have an effect on the acidocalcisomes (ACs), the major Ca^2+^ storage organelles in the cells. We visualized the ACs by immunofluorescence microscopy using the anti- vacuolar proton pyrophosphatase (VP1) antibody (Fig. 6A) (Rodrigues et al., 1999). Additionally, the DNA and cell morphology were visualized by means of DAPI and phase-contrast images (Fig. 6A). We noticed that upon 6 days of TCTP downregulation, the ACs were enlarged in size and the VP1 signal was no longer observed as small dots but as larger ring-like structures (Fig. 6A). This change in AC morphology occurred in all cell cycle stages. We visualized and confirmed this difference through images acquired with super resolution microscopy and transmission electron microscopy (TEM) (Fig. 6B,C). In immunofluorescence microscopy, we counted the total number of ACs in non-induced and induced cells (*n*=10), and for each cell scored them as being dot-like or ring-like shaped. We noticed that, in induced cells, not only was the total number of ACs per cell reduced from an average of 21 to an average of 14, but also the number of ring-like ACs per cell increased up to 4-fold after 6 days of induction (Fig. 6D). In the TEM images, we looked at the ultrastructure of the acidocalcisomes before and after TCTP depletion. As previously shown, ACs are recognized as vesicles containing both material that is electron dense and that is not electron dense (Fig. 6C; Rodrigues et al., 1999). The mean area of the acidocalcisomes was measured in random TEM thin sections (*n*=80) from RNAi-induced and non-induced cells. As already seen in the fluorescence microscopy results, the size of the acidocalcisomes increases significantly (Fig. 6E). We also tested whether the organelle size increase might correlate with an increased storage of phosphate in the organelles; however, we were unable to identify any differences by epifluorescence microscopy. Another important storage compartment for calcium is the mitochondrion. In order to check if the depletion of TCTP leads to mitochondrial structure abnormalities we visualized the PCF mitochondria by immunofluorescence microscopy using an antibody targeting the mitochondrial matrix heat shock protein 70 (HSP-70) (Fig. 7). The images showed that upon 6 days of TCTP depletion, accumulations appeared within the mitochondrial network that can also be detected in transmission electron microscopy (Fig. 7A, arrows point to accumulations; Fig. S4). In the vast majority of cases, there was a single accumulation within one cell and it is located predominantly between the nucleus and kinetoplast DNA (kDNA). As shown in Fig. 7A, formation of these mitochondrial accumulations can occur in all stages of the cell cycle. The percentage of cells with ≥1 accumulations continuously increased from 10% in the non-induced cells (*n*=160) to 40% after six days of *TCTP* RNAi induction (*n*=160) (Fig. 7B).

### Specific depletion of TCTP1 or TCTP2 is not rescued through expression of the remaining paralog

**Fig. 8.**
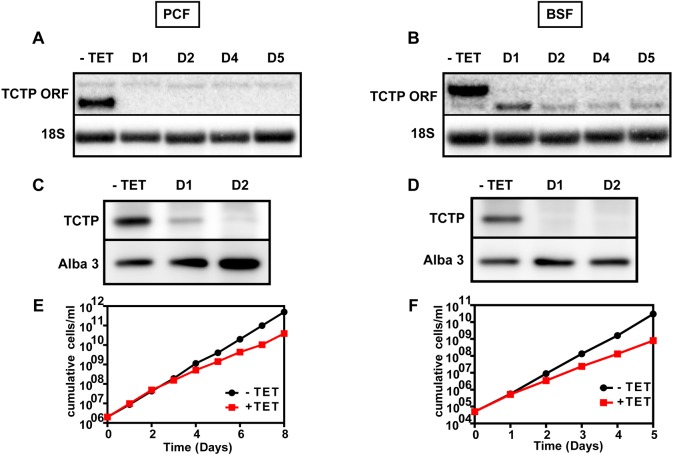
**Effect of TCTP1- and TCTP2-selective depletion on the expression of the remaining paralog in PCF and BSF trypanosomes.** (A,B) Northern blots showing *TCTP1* depletion in PCF and *TCTP2* depletion in BSF upon induction of RNAi targeting the 3′UTRs for the indicated number of days (D1, D2, D4, D5). (C,D) Western blot showing TCTP depletion on day 1 and day 2 following RNAi targeting the 3′UTRs. Alba3 is used as loading control. (E,F) Growth curves of the PCF (*TCTP1* RNAi) and BSF (*TCTP2* RNAi) cell lines in presence or absence of tetracycline (TET).

We were interested to test whether depletion of the life-cycle-specific TCTP can be rescued/compensated for by the cell through expression of the *TCTP* paralog. For this, we used RNAi specifically targeting the *TCTP1* 3′UTR in the PCF and the *TCTP2* 3′UTR in the BSF. The RNA levels of each paralog were detected by northern blot analysis (Fig. 8A,B). Following 1 day of RNAi induction, the levels of *TCTP1* mRNA in the PCF were below the detection limit. No change was observed in the very low levels of *TCTP2* mRNA. In BSF parasites, the mRNA was depleted below detection after 1 day of RNAi induction. This was accompanied by an increase of ∼50% in the levels of *TCTP1* mRNA, however this increase was not maintained during the next time points analyzed. In both life cycle stages, the TCTP protein levels were characterized by immunoblots decorated with anti-TCTP1/2 antibody (Fig. 8C,D) demonstrating that the antibody can efficiently detect both paralogs. At 2 days post RNAi induction, TCTP1 protein is barely detectable in the PCF, while in BSF cells TCTP2 is below the detection limit after 1 day of *TCTP2* RNAi. We also checked whether the life-cycle-specific TCTP1 and TCTP2 depletion leads to a growth phenotype in PCF and BSF cells, respectively (Fig. 8E,F). In the PCF, we observed a growth retardation on day 4 post induction, similar to what we detected for the *TCTP1/2* ORF RNAi (see Fig. 5A), while in the BSF the growth phenotype occurred on day 2 post induction.

## DISCUSSION

TCTP has gained significant attention in recent years because it has been recognized as a therapeutic target in prostate, breast and lung cancers. Consequently, more than 300 publications describe the large variety of processes TCTP might be involved in, including apoptosis, cell cycle regulation, stress response, just to name a few (reviewed in Chan et al., 2012a). Surprisingly little is known about the molecular mechanism by which TCTP is involved in these many different processes or how the gene might be regulated.

This is the first report about localization, function and regulation of TCTP in trypanosomes, which belong to the eukaryotic super-group of the Excavates. Many discoveries made in Excavates and especially in trypanosomes, including GPI anchoring (Ferguson and Cross, 1984), RNA editing (Benne et al., 1986), trans-splicing (Sutton and Boothroyd, 1986) and acidocalcisomes (Docampo et al., 1995), have later also been found to be important in the distantly related but more commonly studied eukaryotes like yeast, *C. elegans* and mammalian cells.

We identified a paralog to the already annotated *TCTP* in the *T. brucei* genome to be encoded in tandem. This feature seems to be conserved within the Trypanosomatida, while *Bodo saltans* and *Perkinsela* sp., for example, only contain one ortholog in the current genome annotation (BS72265, *Bodo saltans*; KNH04185, *Perkinsela* sp.). This, together with the fact that the sequence conservation of the two *TCTP* paralogs within the different Trypanosomatida is very high, might point towards a duplication event just prior to the separation of the Trypanosomatida from the other Kinetoplastea.

While the 5′UTRs of the two paralogs in *T. brucei* are identical and the ORFs only contain ten nucleotide polymorphisms, the 3′UTRs are very different in length and sequence. A similar situation has recently been described for two genes encoding cytoskeletal proteins in *T. brucei*; however, there the mechanism of gene expression regulation remained unexplored (Portman and Gull, 2014). For the two *TCTP* paralogs in *T. brucei*, we provide strong evidence that the mechanism by which these two genes are differentially regulated is based on the different 3′UTR sequences, providing a prominent example of post-transcriptional regulation in *T. brucei*. Interestingly, the first 160 nt of the *TCTP2* 3′UTR seem to be sufficient to increase stability of the *CAT* reporter transcript in the BSF cells even in the context of the entire *TCTP1* 3′UTR (see Fig. 3G). Thus, there is an element in the first 160 nt of the *TCTP2* 3′UTR that is able to override the destabilization features of the *TCTP1* 3′UTR in BSF cells. Even more surprising is the finding that the same 160 nt lead to a destabilization in the PCF cells, thus overriding at least partially the stabilization mediated by the *TCTP1* 3′UTR. If the stabilization and destabilization effects are a consequence of the same element within the 160 nt or if there are multiple elements remains to be investigated.

The difference in half-life of the two *TCTP* transcripts (Fig. 2D), can at least partially be attributed to the different growth rates of the two life cycle stages. Interestingly, the aspect of two different 3′UTRs in *TCTP* transcripts is also found in other species. In humans and rabbits, for example, the *TCTP* gene is transcribed as two mRNAs that only differ in the length of their 3′UTR as a result of differential polyadenylation (Thiele et al., 2000). Both mRNAs are co-expressed in almost all tissues. Interestingly, the *TCTP* transcript with the shorter UTR is often times more abundant than the long UTR counterpart, however so far no function has been assigned to this finding (Thiele et al., 2000). It would be interesting to test if this ratio would change upon modification of the respective 3′UTRs in humans, and whether this change in ratio would further lead to the cellular impairment described in the many TCTP studies. Our results clearly demonstrate a function for the 3′UTRs in the stability of the mRNAs; however, they do not exclude that the different UTRs might also have additional functions, a possibility that has previously been described albeit for the entire *TCTP* mRNA. Bommer and colleagues suggest that the *TCTP* mRNA is a highly folded mRNA that can bind to and activate the double-stranded RNA (dsRNA)-dependent protein kinase (PKR), an important regulator of translation (Bommer et al., 2002). Thus, one could speculate that the different *TCTP* 3′UTRs might also be involved in the specific biology of the two different life cycle stages.

The cytoplasmic localization of TCTP1 in *T. brucei* is similar to what has been described for most organisms (Acunzo et al., 2014; Rinnerthaler et al., 2006), and was recently confirmed by a tagging screen of the TrypTag consortium (Dean et al., 2017) on the TriTrypDB website. However, the TrypTag study additionally identified TCTP1 at the flagellum. We believe the difference in localization could be due to the difference in the protein tag. While we expressed TCTP fused to rather small (≤5 kDa) peptides, the TrypTag consortium relied on the much larger mNeonGreen (26.6 kDa, Shaner et al., 2013). Furthermore, the expression levels of the mNeonGreen tagged TCTP seem to be higher, which might also lead to additional localizations. Finally, it is unclear which of the pararlogs was tagged in the TrypTag screen so it might be that expression of the BSF TCTP2 in the PCF cells leads to a different localization. One proteomics study has also identified a small number of TCTP1 peptides in isolated flagella (Subota et al., 2014), while it was not detected in two other proteomics studies of the flagellum (Oberholzer et al., 2011; Zhou et al., 2010). Although, we cannot exclude that TCTP has localizations outside the cytoplasm, we think that the biochemical evidence convincingly shows a cytoplasmic localization with the tagged and the native protein. As mentioned above, a large number of functions are ascribed to TCTP in the different model systems, and very little is known about the mechanisms by which the functions are performed. We used RNAi and efficiently depleted the *TCTP* mRNA in procyclic trypanosomes. While TCTP depletion led to a growth defect, it seemed to not affect all cells in the population. After several days of RNAi induction, up to 40% of cells showed a distinct tadpole-like morphology phenotype, while the majority of the cells had a wild-type appearance. One possible explanation for the growth phenotype is that the tadpole-shaped cells are a dead end and cannot further divide. However, since we rarely observed dying cells it is more likely that the tadpole-like cells simply require more time to remodel their cytoskeleton before they can re-enter the cell cycle.

During cytokinesis of PCF parasites, the posterior part of the cell, including the new flagellum, is inherited to one daughter while the anterior region with the old flagellum is inherited to the other daughter cell. Consequently, the central part of the cell has to be remodeled such as it can give rise to a new posterior for the daughter cell with the old flagellum and a new anterior for the daughter with the new flagellum (Wheeler et al., 2013). We hypothesize that this remodeling process, and more specifically the posterior end processing of the new daughter cell with the old flagellum, is impaired through the loss of TCTP1 (Fig. 5). Alternatively, it could also be that the posterior end development of the daughter cell with the new flagellum that is delayed and thus contributes to the decrease in growth. The involvement of TCTP1 in a microtubule-related process is supported by the findings in other systems where TCTP has been demonstrated to be a microtubule-binding protein and to be involved in the formation of the mitotic spindle (Gachet et al., 1999). Interestingly, we also detected a change in mitotic spindle staining in the PCF cells upon RNAi-mediated depletion of TCTP (Fig. S5); however, neither our imaging data nor our biochemical data support any direct involvement of TCTP with cytoskeletal structures. Furthermore, we did not detect any obvious changes in the microtubule corset through staining of the tyrosinated ±-tubulin with YL1/2 antibody or by evaluating TEM sections, and can likely exclude that TCTP is involved in the biogenesis of other microtubule-rich organelles, like the basal body or flagellum, since these structures were not visibly affected in PCF *TCTP1/2* RNAi knockdown cells (Figs S6 and S7).

TCTP downregulation in PCF also had an effect on the morphology and the number of acidocalcisomes, the main Ca^2+^ and polyphosphate storage organelles, which were first discovered in trypanosomes and later in other organisms (Docampo and Moreno, 2011; Moreno et al., 1992). Upon downregulation of TCTP, we observed that the number of acidocalcisomes per cell decreased, while the size of the remaining organelles almost doubled. We tested whether this size increase could be due to an accumulation of polyphosphates, but did not detect any changes by epifluorescence microscopy. We also detected changes in mitochondrial morphology. Since the organelle morphology and growth defect occur during the same time frame, we cannot conclude at this point whether one is a secondary effect of the other. Future experiments will address this question, as well as how TCTP is involved in Ca^2+^ homeostasis.

We further investigated whether the depletion of the life cycle-specific paralog could be rescued by the expression of the other one. While we observed no changes in *TCTP2* mRNA level in the PCF, we observed an initial upregulation of *TCTP2* mRNA in the BSF. However, this was only detected 24 h post *TCTP1* RNAi induction and was not reflected in protein levels, which were below detection limit at this time point. Additionally, in both the PCF and BSF a growth retardation phenotype was detected.

In conclusion, this study, for the first time, characterizes the universally conserved TCTP protein in the Excavates and attributes a function to the different 3′UTRs of *TCTP*, a feature that is seen in many different phylogenetic groups. Our experiments clearly demonstrate that the 3′UTRs are the elements responsible for the differential expression of *TCTP1* and *TCTP2* paralogs in the BSF and PCF trypanosomes. Similar to what has been described previously in other phylogenetic groups, TCTP seems to be involved in a variety of functions and, based on the acidocalcisome and mitochondrial phenotypes, we add Ca^2+^ homeostasis to the list of processes that TCTP is potentially involved in.

## MATERIALS AND METHODS

### Trypanosome cell lines and culturing

For RNAi and gene tagging experiments, we used transgenic *T. brucei* bloodstream (New York single marker, NYsm) and procyclic (29-13) cell lines co-expressing T7 RNA polymerase and a tetracycline repressor (Wirtz et al., 1999). The BSF cells were cultured at 37°C and 5% CO_2_ in HMI-9 medium supplemented with 10% fetal calf serum (FCS) (Hirumi and Hirumi, 1989) in the presence of 2.5 µg/ml geneticin (G418). The PCF cells were maintained at 27°C in SDM-79 medium with 10% FCS (Brun and Schönenberger, 1979) in the presence of 15 µg/ml geneticin and 25 µg/ml hygromycin. For the CAT assay, we used wild-type *T. brucei* 427 BSF expressing VSG221 (MITat 1.2) and wild-type 427 PCF (Brun and Schönenberger, 1979; Cross and Manning, 1973). The cell lines were maintained in the same media as the transgenic lines, but in absence of the antibiotics. The cell lines were obtained from the established collection of the Institute of Cell Biology, University of Bern, Bern, Switzerland.

### Plasmid constructs and transfection

For inducible RNAi against *TCTP1* and *TCTP2* mRNAs, we used a pLEW100-based stem-loop plasmid (Mani et al., 2015; Wirtz et al., 1999) where an insert of 512 bp targeting the full ORF sequence of the *TCTP1* gene was integrated. For the inducible RNAi against *TCTP1* or *TCTP2* 3′UTRs, one insert targeting the specific 1–489 bp of the *TCTP1* 3′UTR and one insert targeting the specific 17–520 bp of the *TCTP2* 3′UTR were used. The constructs were linearized with *Not*I, and 10 µg were transfected in NYsm BSF and/or 29-13 PCF by electroporation. The positive clones were selected with blasticidin (5 µg/ml in BSF and 10 µg/ml in PCF). Induction of RNAi was achieved by addition of 1 µg/ml tetracycline. For the C-terminal tagging, one allele of *TCTP1* in PCF was *in situ* tagged with a triple hemagglutinin (HA) epitope (Oberholzer et al., 2006). For inducible N-terminal c-Myc tagging, the full ORF plus the first 21 nt from the 3′UTR of *TCTP1* were amplified by PCR and cloned in pJM-2 vector (a gift from André Schneider, Department of Chemistry and Biochemistry, University of Bern, Switzerland; Mani et al., 2015). Upon transfection (as described above) the clones were selected with puromycine. Expression was induced by addition of 1 μg/ml tetracycline. For the CAT activity assay, the endogenous 3′UTR of *TCTP1* (1–505 nt) or *TCTP2* (1–944 nt) was cloned into pHD2169 vector downstream of the *CAT* ORF, between the *Bam*HI and *Sal*I restriction sites (Droll et al., 2013, a gift from Christine Clayton, ZMBH, University of Heidelberg, Germany). The plasmid was linearized by *Not*I digestion at the β-tubulin-targeting sequence. The constructs were transfected in wild-type BSF and PCF cells (as described above) and the clones were selected with G418. To determine the sequences of the *TCTP2* 3′UTR responsible for the BSF-specific expression, truncated versions of *TCTP2* 3′UTR (Δ1–160; Δ1–376 and Δ1–576) were cloned into the pHD2169 using the Gibson assembly master mix (NEB) and transfected in wild-type BSF. The clones were selected with G418.

### Northern blotting and RNA analysis

For northern blot analysis, total RNA was extracted from trypanosomes by re-suspending 10^8^ trypanosomes in 1 ml RiboZol™ (Amresco). The RNA was purified by phenol/chloroform extraction, precipitated with ethanol and, unless directly used, stored at −20°C. 8–10 µg RNA was resolved in 1.4% agarose gels and transferred overnight onto nylon cellulose membranes (Millipore) by capillary blotting. The membranes were incubated overnight with radioactively labeled (α-[^32^P]dCTP), PCR-generated probes using a Random Primed DNA Labeling Kit (Roche). For normalization, the blots were re-probed for 18S ribosomal RNA (rRNA). The 18S rRNA probe was generated by T4 PNK labeling of an 18S oligonucleotide with γ-[^32^P]ATP. The probes for the ORF and 3′UTRs of *TCTP1* and *TCTP2* were generated by amplifying the ORF or specific fragments of the 3′UTRs after the stop codon: 14–488 nt for *TCTP1* and 6–606 nt for *TCTP2* using the genomic DNA as template. The CAT probes were generated by amplification of the *CAT* ORF (13–436 nt) using pHD2169 as template. For the mRNA decay analysis, the transcription was blocked by addition of ActD (10 µg/ml) and total RNA was isolated from the cells (as described above) at different time points after addition (0 min, 10 min, 40 min, 90 min, 240 min, 420 min and 600 min). Three biological replicates were performed. GraphPad Prism was used to plot the mean relative mRNA abundances at each time point (normalized to 18S RNA) and to calculate the mRNA half-lives.

### Generation of polyclonal anti-TCTP antibody

The full-length ORF of *TCTP1* was cloned into pHIS-parallel-1 vector, which allows the expression of TCTP1 as a fusion protein with the HIS_6_ peptide (Sheffield et al., 1999). Expression of the fusion protein was induced for 2 h in *E. coli* BL21 paplac strain at an optical density at 600 nm (OD_600_)=0.8 by addition of 1 mM isopropyl β-D-1-thiogalactopyranoside (IPTG). Next, the bacteria were kept on ice and re-suspended in lysis buffer [50 mM NaH_2_PO_4_ pH 8.00, 300 mM NaCl, 5 mM Imidazol, 10% (v/v) glycerol, protease inhibitors], sonicated and spun down at 9500 ***g*** for 30 min at 4°C. The supernatant containing the proteins was loaded into Ni-charged IMAC resin columns (Bio-Rad) and the His_6_ tag fusion TCTP1 protein was purified by immobilized metal affinity chromatography in elution buffer (50 mM NaH_2_PO_4_ pH 8.00, 300 mM NaCl, 20 mM Imidazol) and used to produce rat polyclonal antibody (Eurogentec, Belgium).

### Western blotting

For western blotting, trypanosome pellets were washed in phosphate-buffered saline (PBS, pH 7.2) and re-suspended in standard Laemmli buffer (LB) (10^6^ cells in 15 µl). For the digitonin fractionation, the cells were washed once in PBS, then the pellets were re-suspended in SoTE buffer (0.6 M sorbitol, 2 mM EDTA, 20 mM Tris-HCl pH 7.5) containing 0.025% digitonin and incubated on ice for 5 min. Next, the cell fractions were separated by differential centrifugation at 8000 ***g*** for 5 min at 4°C. This gives a supernatant fraction enriched in cytosolic proteins and a pellet enriched in organelles and organelle-bound proteins. Both fractions were lysed in LB, boiled for 5 min at 95°C, cooled on ice for 5 min and loaded on 10% or 12% SDS-polyacrylamide gels (10^6^–10^7^ cells per lane) before being subjected to western blotting. Next, the proteins were transferred onto PVDF Immobilon-P membranes (Millipore) by using a BioRad wet blotting system and blocked for 1 h at room temperature in 10% skimmed milk or bovine serum albumin (BSA) solution in PBST (PBS plus 0.1% Tween-20). The primary antibodies were incubated for 1 h at room temperature or overnight at 4°C in PBST. In this study we used rat-polyclonal anti-TCTP (1:50, produced for us by Eurogentech), rabbit anti-ATOM (1:10,000, Pusnik et al., 2011), mouse anti-EF1α (1:10,000, cat. no. sc-21758, Santa Cruz Biotechnology), rabbit anti-Myc (1:1000, Sigma) and rabbit anti-Alba3 (1:1000, Mani et al., 2011) antibodies. After washing the primary antibody by incubating the membranes three times, for 10 min each time, in PBST, the membranes were incubated for 1 h at room temperature with the secondary antibody. Secondary antibodies were: horseradish peroxidase (HRP)-conjugated rabbit anti-rat-IgG, rabbit anti-mouse-IgG and swine anti-rabbit IgG (all 1:10,000, Dako). For the chemiluminescent detection, the SuperSignal system (Pierce) was used, and images were acquired with an Amersham Imager 600.

### Immunofluorescence and microscopy

For immunofluorescence, BSF or PCF cells were harvested by a slow centrifugation (2000 ***g*** for 5 min), washed once in PBS and then fixed on slides for 4 min with 4% paraformaldehyde (PFA) in PBS. The cells were permeabilized for 5 min with 0.2% Triton X-100 in PBS and blocked for 30 min with 4% BSA in PBS. The cells were incubated for 1 h in a wet chamber with primary antibody diluted in 4% BSA in PBS, washed three times in PBST and incubated again in dark wet chambers with secondary antibodies diluted in 4% BSA in PBS. The cells were mounted with ProLong^®^ Gold Antifade Mountant with or without DAPI (Invitrogen). Images were acquired with Leica DM 5500 fluorescent light microscope and/or Leica SP8 Confocal Microscope System with STED and deconvolved with Leica LAS AF and Huygens software, respectively. The primary antibodies used in this study were: rat anti-PFR (1:1000, Kohl et al., 1999), rat anti-YL1/2 (1:2000; Kilmartin et al., 1982), mouse anti-KMX (1:500, Sasse and Gull, 1988), mouse anti-mtHSP70 (1:2000, Klein et al., 1995), rabbit anti-VP1 (1:2000, Rodrigues et al., 1999), rabbit anti-HA (1:1000, cat. no. H6908, Sigma) and rabbit anti-Myc (1:1000, cat. no. C3956, Sigma). Secondary antibodies were goat anti-rabbit IgG, goat anti-rat IgG, goat anti-mouse IgG conjugated to fluorophores Alexa Fluor^®^ 488, Alexa Fluor^®^ 594 (1:1000, Invitrogen).

### Electron microscopy

To prepare samples for electron microscopy, non-induced and induced (day 6) PCF trypanosomes were pelleted by centrifugation (3325 ***g*** for 5 min), washed once in PBS and submerged with a fixative prepared as follows: 2.5% glutaraldehyde (Agar Scientific, Stansted) in 0.15 M HEPES (Fluka) with an osmolarity of 684 mOsm and adjusted to a pH of 7.41. The cells remained in the fixative at 4°C for at least 24 h before being further processed as previously described in Trikin et al. (2016). The images were analyzed with ImageJ.

## Supplementary Material

Supplementary information
