# Supplementary material for: Distinct 3′ UTRs regulate the life-cycle-specific expression of two TCTP paralogs in Trypanosoma brucei
Source: J Cell Sci. 2018 May 10;131(9):jcs206417. doi: 10.1242/jcs.206417 (PMC5992589; doi:10.1242/jcs.206417)
Supplement: Supplementary information [file joces-131-206417-s1.pdf]

Supplementary information

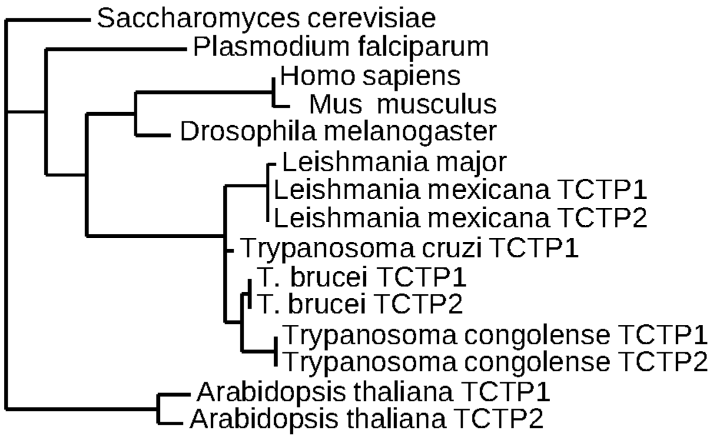

Fig S1. Phylogenic tree of TCTP based on amino acid sequences of representative species. *L. major* (Q4QAI0), *L. mexicana* (E9AWZ6 and E8NHJ5), *T. cruzi* (V5DBM6), *T. congolense* (G0USS4 and G0USS5) and other well-studied organisms *S. cerevisiae* (P35691), *P. falciparum* (Q8I3Z5),.*M. musculus* (P63028), *H. sapiens* (P13693), *D. melanogaster* (Q9VGS2) and *A. thaliana* (P31265 and Q9M9V9).

Journal of Cell Science • Supplementary information

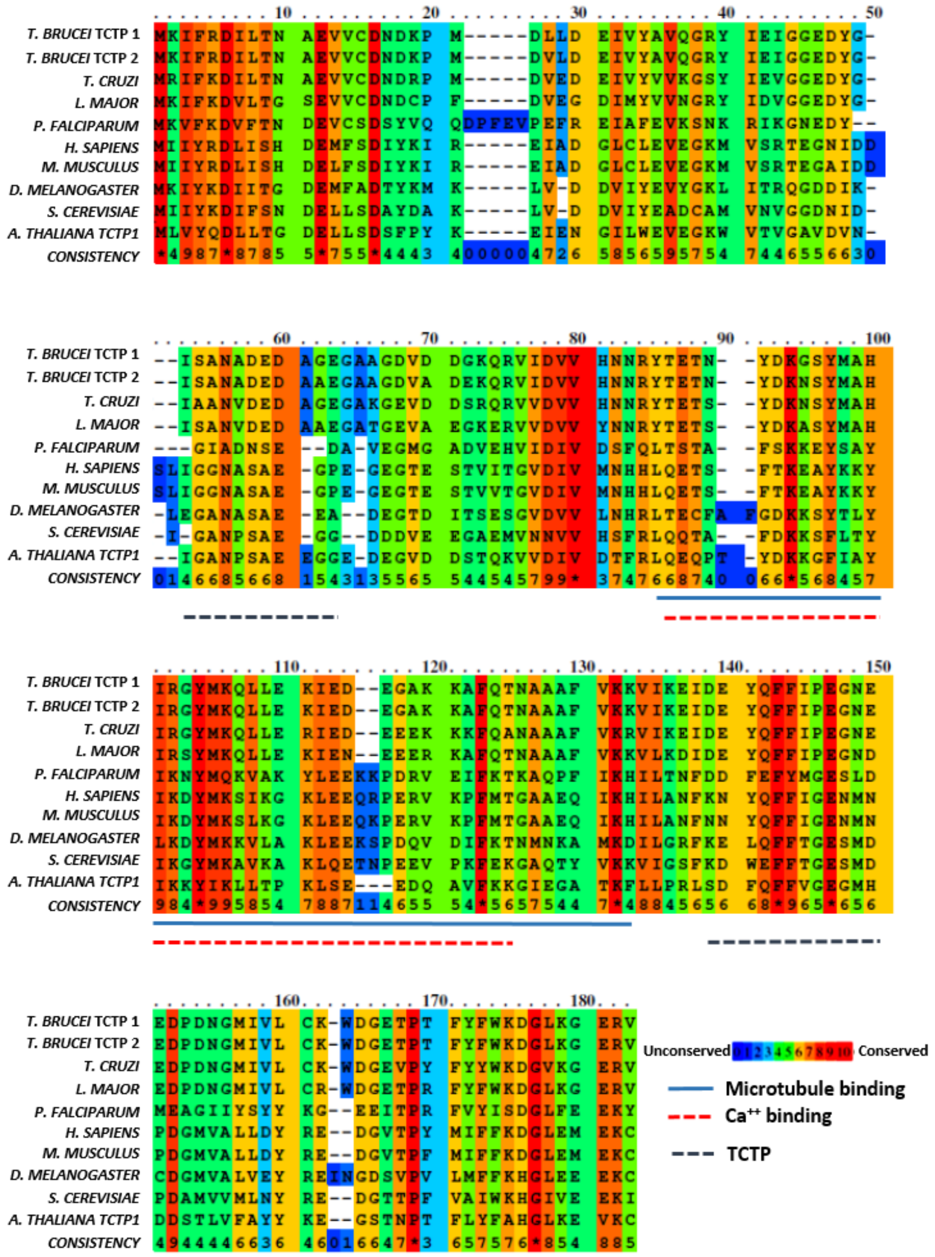

Fig S2. TCTP sequence comparison and predicted domains in *T. brucei* and representative species. The alignment and conservation scoring of amino acid sequence was done using Praline. The amino acid count is based on the *T. brucei* TCTP1 sequence. The color-coded scheme works from 0 for the least conserved alignment position to 10 for the most conserved alignment position. Identical residues in all the aligned species are shown by an asterisk Displayed are the characterized domains in other organisms: the microtubule binging domain ((Gachet et al., 1999), blue line), the Ca^{2+} binding domain ((Kim et al., 2000) dashed red line) and the two TCTP signature domains ((Bommer and Thiele, 2004), dashed black line)

Journal of Cell Science • Supplementary information

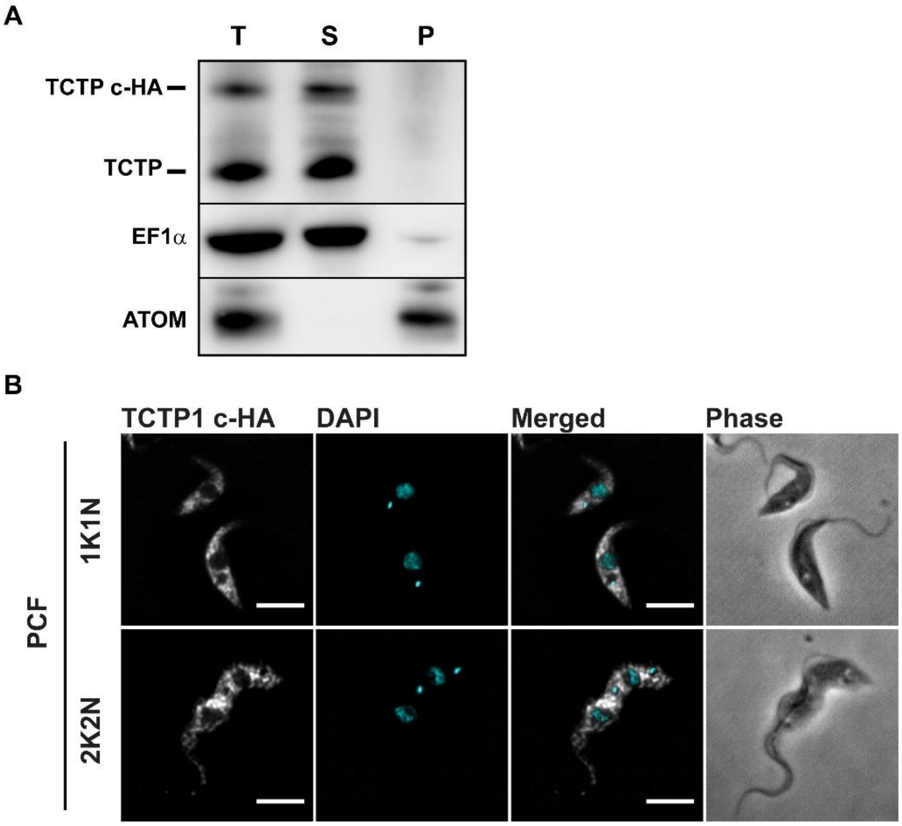

Fig S3. Localization of C-terminal in-situ tagged TCTP1 in procyclic trypanosomes. TCTP1 was tagged with and visualized Trypanosomes expressing triple HA in-situ tagged TCTP1 were lysed with 0.025% digitonin and the cell extracts were fractionated by differential centrifugation. Total cellular extract (T), supernatant (S) and pellet (P) fractions were analyzed by western blot decorated with antibodies against TCTP, ATOM and EF1α. (B) C-terminally tagged TCTP1 was visualized by immunofluorescence microscopy with anti-HA antibody (white). The nuclear and mitochondrial DNA are detected by DAPI (blue). Scale bars: 5 µm

Journal of Cell Science • Supplementary information

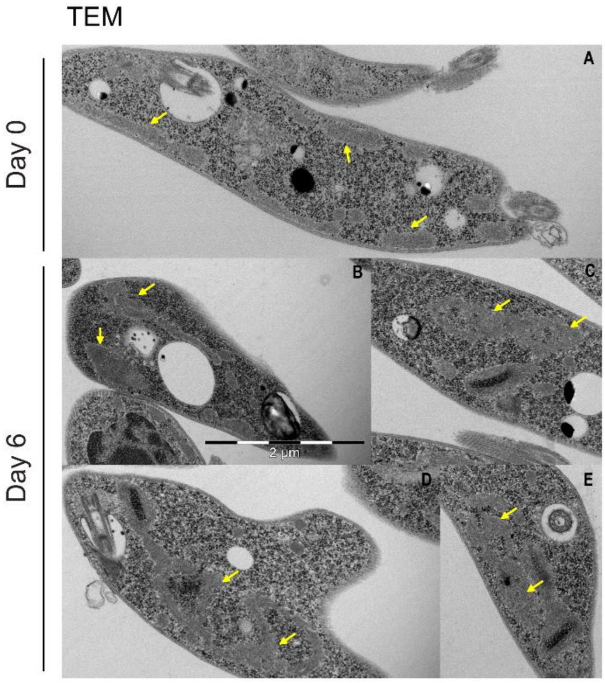

Fig S4. Detection of mitochondrial accumulation by TEM following TCTP downregulation. Representative images of mitochondria ultrastructure by transmission electron microscopy. Arrowheads point to accumulations.

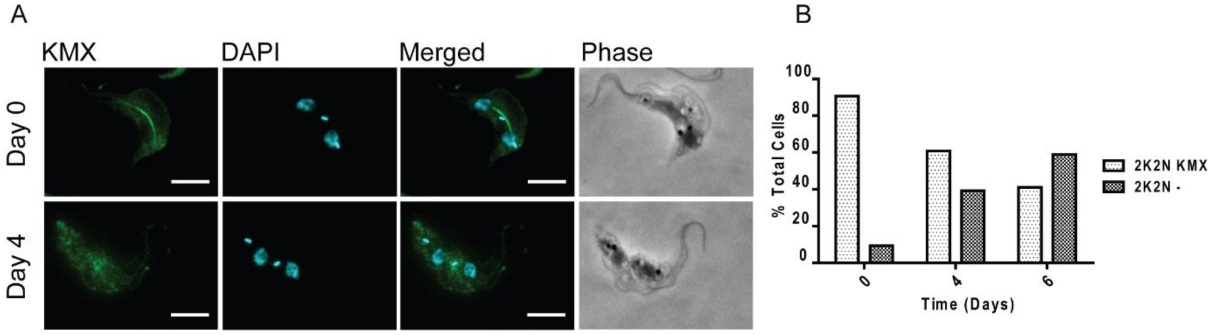

Fig S5. KMX staining of the mitotic spindle is partially lost upon *TCTP1/2* RNAi in PCF. (A) Immunofluorescence staining of uninduced and induced 2K2N cells. Staining for β-tubulin (KMX, green) and for the nucleus and kDNA (DAPI, cyan) is shown. (B) Quantification of 2K2N cells showing KMX staining during the course of *TCTP1/2* RNAi induction (n≥40). Scale bars: 5 µm

Journal of Cell Science • Supplementary information

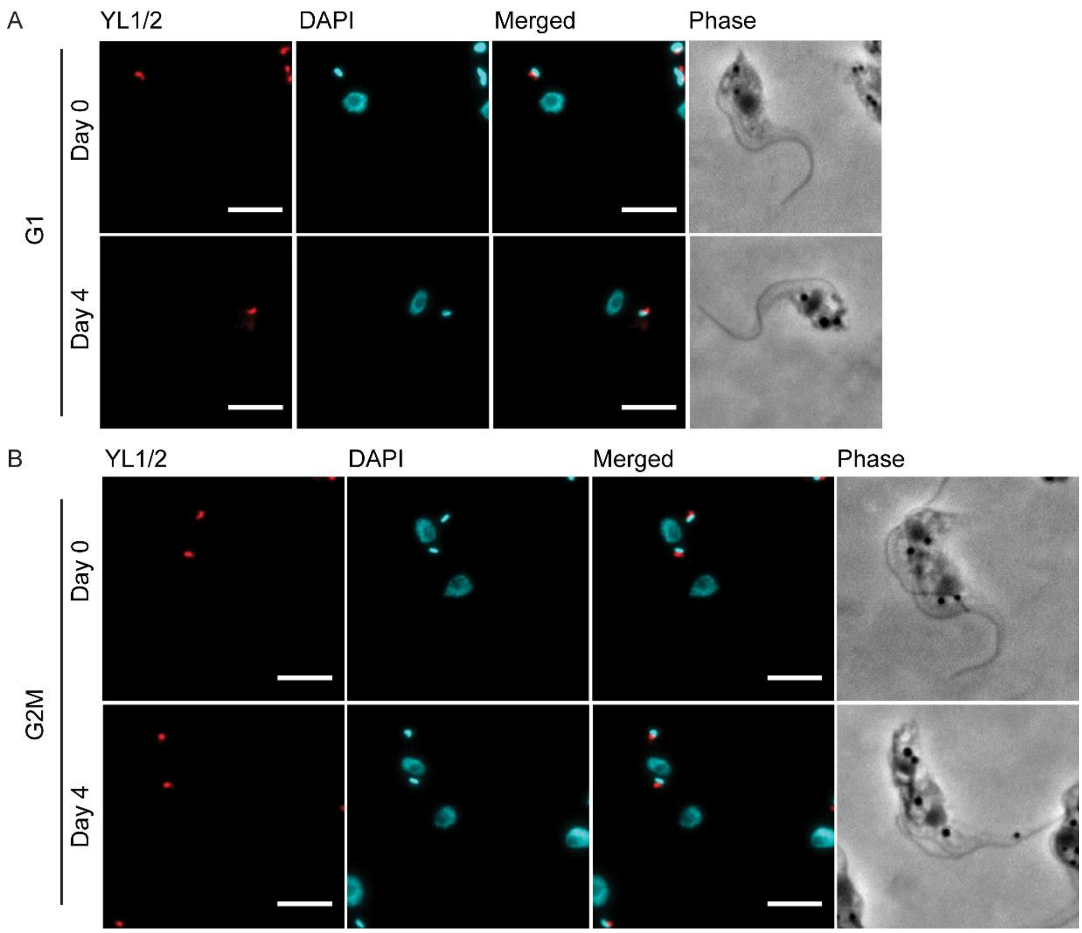

Fig S6. Downregulation of TCTP in procyclic trypanosomes does not impair the basal body and its segregation during the cell cycle. Non-induced (Day 0) and induced (Day 4) procyclic trypanosomes were stained for the mature basal body (YL 1/2, red) and nuclear and kinetoplast DNA (DAPI, cyan). Morphology of the cells is shown in phase contrast images (grey). Representative images of G1 (A) and G2M (B) cells are shown. Scale bar: 5 μm.

Journal of Cell Science • Supplementary information

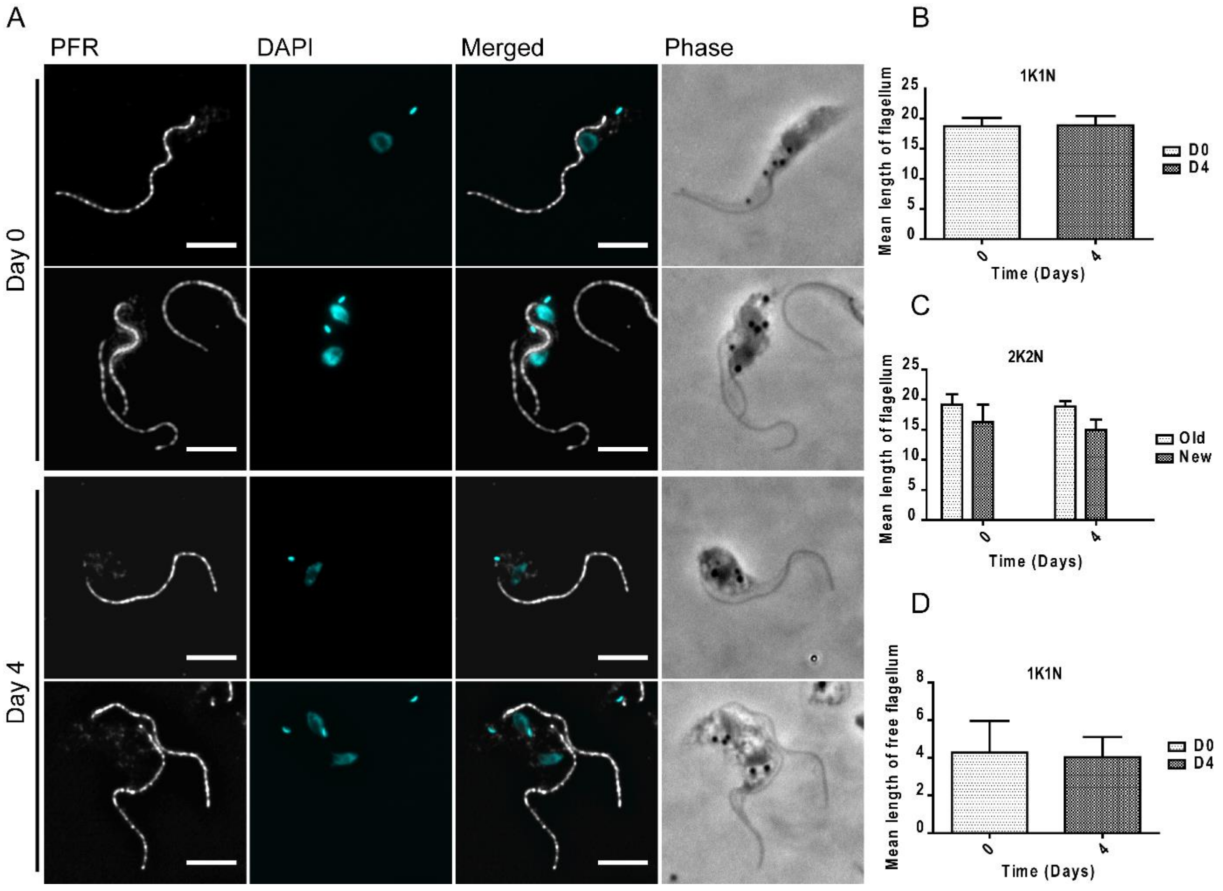

Fig S7. Flagellum length analysis of wild type and *TCTP1/2* RNAi procyclic cells. (A) Immunofluorescence images non-induced and day 4 induced procyclic trypanosomes with the PFR antibody (white) and DAPI (cyan). Cell morphology is shown with phase contrast (gray). (B-C) Length measurements of PFR stained flagella in 1K1N cells (n=25) and 2K2N cells (n=10). (D) Measurements of free flagellum length in 1K1N of cells based on phase contrast images (n=16). Scale bars: 5 µm.

Journal of Cell Science • Supplementary information
